# HDAC7: a promising target in cancer

**DOI:** 10.3389/fonc.2024.1327933

**Published:** 2024-02-28

**Authors:** Cui Liu, Dan Zheng, Xuan Pu, Sijun Li

**Affiliations:** Department of Otolaryngology-Head and Neck Surgery, Affiliated Hospital of North Sichuan Medical College, Nanchong, China

**Keywords:** HDAC7, cancer, signaling pathway, molecular mechanism, inhibitors

## Abstract

Histones have a vital function as components of nucleosomes, which serve as the fundamental building blocks of chromatin. Histone deacetylases (HDACs), which target histones, suppress gene transcription by compacting chromatin. This implies that HDACs have a strong connection to the suppression of gene transcription. Histone deacetylase 7 (HDAC7), a member of the histone deacetylase family, may participate in multiple cellular pathophysiological processes and activate relevant signaling pathways to facilitate the progression of different tumors by exerting deacetylation. In recent years, HDAC7 has been increasingly studied in the pathogenesis of tumors. Studies that are pertinent have indicated that it has a significant impact on the growth and metastasis of tumors, the formation of the vascular microenvironment, and the emergence of resistance to drugs. Therefore, HDAC7 could potentially function as a potent predictor for tumor prognosis and a promising target for mitigating drug resistance in tumors. This review primarily concentrates on elucidating the structure and function of HDAC7, its involvement in the development of various tumors, and its interplay with relevant signaling pathways. Meanwhile, we briefly discuss the research direction and prospect of HDAC7.

## 1 Introduction

Histones, which are present in both eukaryotic chromatin and prokaryotic basic proteins, could associate with DNA to form nucleosomes and play a regulatory role in gene expression within the cell nucleus (1). When engaged in histone modification, HDACs catalyze lysine deacetylation to compact chromatin and suppress the transcription of associated genes (2). HDAC7, one of the 18 members in the HDACs family, has been linked to significant functions in various physiological and pathological processes. Accumulating studies have shown that HDAC7 is not only involved in critical physiological processes such as immunity (3, 4), osteogenesis (5), angiogenesis (6), and energy metabolism (7), but also in the development of various diseases including tumors.

To regulate specific signaling pathways, HDAC7, which is part of the HDAC group, causes the deacetylation of β-catenin and STAT3 (8, 9). Abnormal expression of HDAC7 is associated with the prognosis of various tumors and is involved in pathophysiological processes such as tumor cell proliferation, migration, apoptosis, and angiogenesis in the tumor microenvironment. It has a vital function in the development and metastasis of tumors. In oncology therapy, the aberrant expression of HDAC7 enables the occurrence of drug resistance in colorectal cancer (10) and breast cancer (11). Vorinostat, a pan-HDAC inhibitor targeting in HDAC7 and other HDACs, has been used for the treatment of refractory cutaneous T-cell lymphoma (12). Therefore, HDAC7 has the potential to serve as a prognostic indicator and target for tumor treatment. The study of HDAC7 in tumors is growing, but there is a scarcity of comprehensive evaluations regarding the involvement of HDAC7 in tumors. This paper will review the structure, function, and role of HDAC7 in cancer.

## 2 HDACs and HDAC IIa

**Figure 1:**
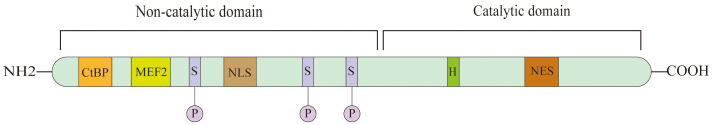
Schematic view of HDAC7, HDAC7 contains two domains which is catalytic domain and non-catalytic domain. Serine(S) residue regulate HDAC7 nuclear- cytoplasmic shuttling; Histidine(H) residue is essential for enzymatic activity; MEF2 is a bind site for members of MEF2 transcription factor family; NLS, nuclear localization signal; NES, nuclear export signal; CtBP is a binding site for с terminal binding protein.

There are 18 kinds of HDACs variations in humans, categorized into four classes (I-IV) according to their similarity in sequence with the yeast counterparts Rpd3, HadI, and Sir2 (13). In terms of structure, they have a catalytic domain at the C-terminal and a regulatory domain at the N-terminal. The catalytic domain of HDACs from classes I, II, and IV includes preserved amino acid residues that bind Zn2+ through chelation. HDACs of class III contain residues that can bind with nicotinamide adenine dinucleotide, in addition to those mentioned above. One distinguishing feature of Class IIa HDACs is the presence of a nuclear export signal (NES) in the C-terminal region, while the N-terminal region is relatively larger and less conserved (**Figure 1**). The N-terminal region contains a nuclear localization signal (NLS) and includes a domain that interacts with members of the myocyte enhancer factor 2 (MEF2) transcription factor family (14). Class IIa HDACs are critical regulatory factors for specific developmental and differentiation processes (15).

The modulation of the interaction between HDAC7 and chaperone proteins, along with the regulation of protein stability, is significantly influenced by phosphorylation, similar to other class IIa HDACs (16). Specifically, the residues S181, S155, S358, and S486 in HDAC7 are of utmost importance in these processes (17). It is worth mentioning that CaMKI can alter the last three locations (18), whereas PKD can phosphorylate all four positions (17). Interestingly, PP2A facilitates the dephosphorylation of the four 14–3-3 binding sites in HDAC7, without showing any preference for any specific site among them (19).

## 3 The structure and functions of HDAC7

HDAC7 is a protein that contains 912 amino acids and is encoded by the HDAC7 gene on human chromosome 12 (20). This protein is abundant in the cardiac, placental, pancreatic, and smooth muscle tissues, and can be located within the cytoplasm or nucleus (21). HDAC4, 5, 7, and 9, which are member of the group of 11 classical HDACs that rely on zinc, function as class IIa HDAC enzymes. These enzymes play a role in controlling cellular and developmental processes using both enzymatic and non-enzymatic mechanisms (22). Similar to other traditional HDACs, class IIa HDACs have a cylindrical binding pocket for substrates, which contains the zinc cofactor at its end (23). Most classical HDACs (24) rely on a tyrosine and two histidine residues within the active site to perform their enzymatic activity. Nevertheless, in class IIa HDACs, the tyrosine is replaced by a histidine, leading to a notable reduction in the enzymatic function for removing acetyl groups from lysine on particular peptide substrates (25). Recent studies have demonstrated that certain stimuli elicit the activation of class IIa HDAC enzymes within the cytosol of cells (26). There is a possibility that a connection with HDAC3, a type I HDAC, might contribute to enabling certain enzyme-mediated functions (27). Additionally, it is conceivable that class IIa HDACs may engage in alternative post-translational modifications beyond lysine deacetylation.

**Figure 2:**
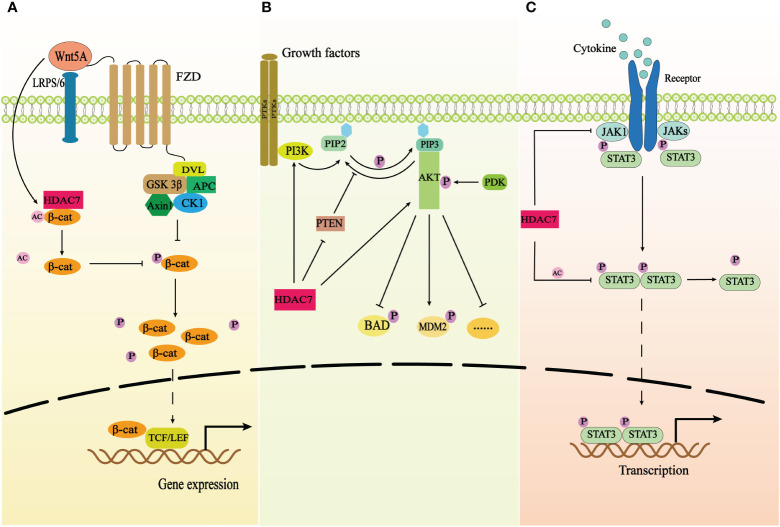
The correlation of HDAC7 and three signaling pathways. **(A)** The schematic demonstrates the correlation of HDAC7 and Wnt/β-catenin signaling pathway. HDAC7 facilitates β-catenin redistribution to the nucleus by deacetylating β-catenin and inhibiting its phosphorylation, which results in that the nuclear accumulation of β-catenin binds to TCF/LEF to activate related gene expression. Wnt5A promotes the expression of HDAC7 and exerts synergistic effects on the activation of Wnt/β-catenin pathway. **(B)** The schematic shows the correlation of HDAC7 and PI3K/AKT signaling pathway. HDAC7 can directly promote the expression of PI3K and AKT proteins and decrease the expression of PTEN the repressor of the PI3K/AKT signaling pathway to trigger the activation of the signaling transduction. **(C)** The schematic shows the correlation of HDAC7 and JAK/STAT3 signaling pathway. HDAC7 inhibits phosphorylation of STAT3 by deacetylating STAT3 and downregulating the expression of the protein kinase JAK1 to repress the activation of the signaling pathway.

Similar to other HDAC IIa proteins in terms of structure, the catalytic domain of HDAC7 is located at the C-terminal, while the N-terminal contains the highly conserved regulatory domain. Phosphorylation takes place on the serine residues of N-terminal, specifically Ser155, 178, 181, 321, 344, 446, 479 (20). This phosphorylation facilitates the binding of HDAC7 and 14-3-3 proteins, enabling their involvement in nucleocytoplasmic transmembrane transport (28, 29). Consequently, cells can swiftly and reversibly adjust to new environments as per their requirements (30). Meanwhile, the C-terminal catalytic domain can interact with various molecular chaperones, such as transcription factors USF-1 (31), to regulate multiple cellular functions. HDAC7 can deacetylate both histones and non-histones and regulate multiple biological processes through deacetylation (32), such as organismal immunity (3, 4), angiogenesis (6), embryonic development (6), energy metabolism (7) and other physiological processes. Additionally, HDAC7 plays significant roles in various tumor progression processes through different signaling pathways (**Figure 2**). For example, it hinders the phosphorylation of β-catenin by removing the acetyl group from β-catenin, thus activating the Wnt/β-catenin pathway and facilitating the proliferation and migration of cancer cells (33) (**Figure 2A**).

## 4 The involvement of HDAC7 in cancer

HDAC7 was found to be aberrantly expressed in various tumors, including high expression in malignant tumors such as lung, esophageal, and gastric cancers, and low expression in acute lymphoblastic leukemia derived from B-cell progenitor cells (**Table 1**). The variation in HDAC7 expression was linked to clinicopathological characteristics and unfavorable prognosis in patients, impacting tumor cell proliferation, migration, and apoptosis via distinct pathological mechanisms.

**Table 1:** The role of HDAC7 in cancer.

Cancer type	The expression of HDAC7	Clinical significance	Functions of HDAC7	Related signaling pathways
Lung cancer	High expression	Poor prognosis; advanced TNM stage; and the degree of tumor differentiation	Promote proliferation and metastasis	Wnt/β-catenin signaling (8); STAT3 signaling (9)
Renal cell cancer	High expression	–	Promote tumorigenesis	TGF-β signaling (7)
Glioma	High expression	Poor prognosis	Promote proliferation and inhibit angiogenesis	Wnt/β-catenin signaling (33); STAT3 signaling (34)
Esophageal carcinoma	High expression	Advanced TNM stage; poor prognosis	Promote proliferation and metastasis	Wnt/β-catenin signaling (35)
Choroidal melanoma	High expression	Clinical stage	Promote proliferation and metastasis	C-myc signaling (36)
Nasopharyngeal carcinoma	High expression	Tumor progression; poor prognosis	Promote tumorigenesis	miR-4465-EphA2 signaling (37)
Colorectal cancer	High expression	Poor prognosis	Promote carcinogenesis and chemoresistance	PI3K/AKT signaling (38)
Gastric cancer	High expression	Distant metastasis; poor prognosis	Promote carcinogenesis	PI3K/AKT pathway (39)
Breast cancer	High expression	Recurrence; poor prognosis	Promote tumorigenesis and chemoresistance; inhibit autophagy	–
Pro-ALL	Low expression	Poor prognosis	–	C-myc signaling (40)
Pancreatic cancer	High expression	No clinical significance	Promote proliferation	–
Hepatocellular carcinoma	High expression	Poor prognosis	–	–

### 4.1 Respiratory system tumors

Lung cancer is the prevailing malignant tumor within the respiratory system, with non-small cell lung cancer (NSCLC) emerging as the predominant histopathological type (41). In patients (8), it was discovered that the levels of HDAC7 protein and mRNA were elevated in lung cancer tissues, and this was associated with a poor prognosis, TNM stage, and tumor differentiation degree. In mechanistic studies, it was reported that HDAC7 promoted tumor growth by deacetylating STAT3 and inhibiting tyrosine phosphorylation *in vitro*, but it had no effect on the protein content of STAT3 (9). For another downstream gene, plakoglobin, the combination of HDAC7 and the promoter region of plakoglobin inhibited plakoglobin expression and promoted tumor cell growth and migration (42). OIP5-AS1, an upstream gene of HDAC7, was reported to inhibit miR-140-5p expression, thereby increasing HDAC7 expression and promoting tumor cell metastasis in NSCLC (43). Vascular endothelial progenitor cells were found to be involved in NSCLC neovascularization through HDAC7-mediated cytoskeletal regulation and angiogenic gene transcription in a study of the tumor microenvironment mechanism (44). Recently, it was reported that HDAC7 was stabilized by the deubiquitinase USP10 in NSCLC, preventing its degradation and activating the β-catenin-FGF18 pathway to promote proliferation and migration of NSCLC cells (8). The effect of HDAC7 on β-catenin is similar to that described in esophageal squamous cell carcinoma. Hence, HDAC7 might contribute to the growth and advancement of lung carcinoma via diverse mechanisms and signaling pathway, offering a conceptual foundation for the exploration of anti-cancer medications.

The pathogenesis of nasopharyngeal carcinoma (NPC) is complex. Distinct roles are played by the HDAC family members in NPC. For instance, class I HDACs demonstrate a tumor-suppressing effect (45), whereas HDAC7 shows an oncogenic effect. In NPC tissues, there was a significant overexpression of HDAC7 which led to the upregulation of EphA2. This was achieved by inhibiting the expression of miR-4465, ultimately promoting the proliferation, migration, and invasion of NPC cells *in vitro* (37). Given the molecular mechanism has been shown in previous studies that other members of the miRNA family inhibited HDAC7 expression by degrading HDAC7 mRNA in tumor tissues (46), while the detailed molecular mechanism that miR-4465 acts as an oncogenic factor in NPC has not been investigated, it is unclear whether miR-4465 works in the same manner as other tumors.

### 4.2 Digestive system tumors

The development and advancement of esophageal squamous cell carcinoma (ESCC) are linked to the accumulation of abnormal regulation of oncogenes, resembling numerous other solid tumors (47). HDAC7, one of the abnormally expressed oncogenes, was observed at higher levels in ESCC than in paraneoplastic tissue and normal esophageal mucosal tissue. The abnormal expression of HDAC7 could bind with β-catenin, leading to β-catenin Lys49 deacetylation and subsequent inhibition of β-catenin phosphorylation. Upon entering the nucleus, the β-catenin that is not phosphorylated formed a complex with the TCF4 transcription factor (33). The combination led to the generation of c-myc, which inhibits the synthesis of p21 and p27, hastening the transition from G1 to S phase (48) and ultimately promoting the proliferation of cancerous cells. This study also demonstrated that melatonin, a hormone produced by the pineal gland, could hinder the growth of ESCC tumor by reducing the activity of the HDAC7/β-catenin/c-myc positive feedback loop and suppressing the function of USP10 that is responsible for maintaining the stability of HDAC7 protein (35). An additional mechanism of ESCC has been elucidated, indicating the involvement of HDAC7 in the metastasis of tumor cells mediated by WNT5A (49). These studies reveal tumorigenesis originates from the loss of the body’s constraints on oncogenic factors. Moreover, they provide insights into how the interaction between HDAC7 and the Wnt pathway affects the growth and metastasis of ESCC. Consequently, exploring targeted drug research focused on WNT5A and HDAC7 may offer a promising therapeutic approach for ESCC.

Currently, the long-term survival rate of patients with gastric cancer following conventional treatment remains suboptimal, thereby necessitating the identification of more efficacious targeted therapeutic agents to improve the survival of such patients (50). The overexpression of HDAC7 in gastric cancer tissues compared to paraneoplastic tissues suggests that it could be a potential therapeutic target for gastric cancer. Furthermore, its abnormal expression has been associated with poor prognosis and distant metastasis in patients diagnosed with gastric cancer (39, 51). Mechanism research has shown that miR-489 is expressed in gastric cancer tissues as an upstream gene of HDAC7. Inhibition of miR-489 may enhance the levels of HDAC7 protein and mRNA expression, thereby activating the PI3K/AKT signaling pathway and facilitating the progression of gastric cancer (39). However, the detailed process which HDAC7 activates the PI3K/AKT signaling pathway has not been further investigated.

Colorectal cancer (CRC) ranks among the top three types of tumors in terms of incidence (52), manifesting diverse genetic and clinical characteristics, as well as a multifaceted pathogenesis (53). A significant increase in the HDAC7 gene expression has been revealed in recent studies of rectal biopsies from individuals with colorectal adenomas, in contrast to those without dysplasia (54). In addition, biopsies obtained from patients diagnosed with colorectal cancer (CRC) showed a comparable increase in HDAC7 expression compared to healthy colon mucosa (55). These findings strongly imply that HDAC7 holds promise as a plausible diagnostic and/or prognostic indicator for CRC. HDAC7, which was significantly upregulated in CRC tissues, could reduce histone acetylation in the promoter region of nucleoside concentrate transporter 2 (CNT2), leading to a contraction in chromatin structure and a decrease in CNT2 expression (10). Furthermore, decreased levels of histone acetylation could impede the absorption of cancerous cells. Likewise, miR-489, the upstream gene of HDAC7, was discovered to promote the proliferation and invasion of CRC cells, resembling its function in gastric cancer, throughout the malignant advancement of colorectal cancer cells (46). Moreover, HDAC7 could be regulated by the miR-140-5p upstream gene. Its overexpression increased the protein levels of PI3K and AKT, thereby inducing tumor cell invasion (38). In summary, HDAC7 exerts its oncogenic effects through different mechanisms. Additionally, it inhibits the uptake of nucleoside anticancer drugs by tumor cells, which allows the development of drug resistance in tumor therapy. Therefore, HDAC7 inhibitors can both combat tumors and reduce the development of related drug resistance.

Pancreatic carcinoma is among the most lethal types of cancer, and there is a shortage of reliable indicators to accurately differentiate between noncancerous and cancerous tumors in the pancreas. The protein and mRNA expression of HDAC7 was found to be upregulated in pancreatic cancer tissues, but the abnormal expression of HDAC7 did not exhibit a clear correlation with the clinical characteristics of patients (56). Given that this is a retrospective study, the causal relationship between the upregulation of HDAC7 and tumor progression remains uncertain. However, evidence from previous research suggests that the inhibition of HDAC7 leads to suppressed cell growth in pancreatic cancer cell lines (57), and its abnormal expression is associated with the progression of other digestive system malignancies, such as colorectal (46) and gastric cancer (39). Therefore, it is reasonable to contemplate the upregulation of HDAC7 as a possible contributing factor in the malignant advancement of cancerous tissues. Further investigation is required to establish the exact cause-and-effect connection and elucidate the intricate process by which HDAC7 promotes the advancement of pancreatic cancer.

Notably, HDAC7 exhibits significantly elevated expression levels in hepatocellular carcinoma and is correlated with poor prognosis in patients with this condition (58). However, the specific role of HDAC7 in the advancement of hepatocellular carcinoma remains unclear. It is worth mentioning that repeated liver injury induces the transformation of hepatocytes from steatosis to fibrosis. According to existing research on the pathogenesis of hepatocellular carcinoma, it has been observed that its progression ultimately culminates in the development of hepatocellular carcinoma (59). However, specific research has suggested a notable correlation between the expression of HDAC7 and collagen α-1, a fibrosis-related protein found in the extracellular matrix (60). Consequently, it is plausible to suggest that HDAC7 may potentially contribute to the pathogenic mechanisms underlying chronic liver disease and the subsequent development of hepatocellular carcinoma.

### 4.3 Glioma

Gliomas, originating from the epithelial cells of the central nervous system, are the prevalent tumors within the brain (61). They are highly aggressive and can directly metastasize to adjacent normal brain tissue, and standard treatment cannot achieve a favorable prognosis (62). This refers to an imbalance between histone acetylation and deacetylation regarding the mechanism of glioma development (63). HDAC7, as a histone deacetylase, had an aberrant expression that contributes to glioma development. Compared to normal brain tissue (64), gliomas exhibited an upregulation in HDAC7 mRNA levels. Silencing HDAC7 could increase STAT3 expression levels and promote STAT3 phosphorylation, inhibiting tumor angiogenesis and tumor cell growth (34). HDAC7 could inhibit the phosphorylation of STAT3 by exerting deacetylation and downregulating the expression of the protein kinase JAK1 (34). Another mechanism suggests that the zinc finger protein ZNF326 is highly expressed in gliomas and increases the transcriptional expression of HDAC7 in tumors. In the meantime, HDAC7 hinders the phosphorylation of β-catenin, enabling the entry of unphosphorylated β-catenin into the nucleus and triggering the activation of the Wnt pathway, ultimately contributing to the proliferation and invasion of glioma (33). In summary, HDAC7 promotes tumor angiogenesis and cell growth by blocking the activation of STAT3 and inhibiting β-catenin phosphorylation, which alters its cytoplasmic-nuclear redistribution and contributes to the malignant progression of glioma (62). This coincides with the highly aggressive characteristics of gliomas.

### 4.4 Ovarian cancer

The recurrence rate of ovarian cancer is not well controlled after first-line clinical treatment (65). The latest research indicates that the recurrence, metastasis, and occurrence of resistance to conventional chemotherapy correlates with the existence of tumor stem cells in ovarian cancer (66). HDAC7 was overexpressed in ovarian cancer stem cells and played an important role in maintaining the tumor stem cell phenotype. Therefore, the overexpression of HDAC7 in ovarian cancer stem cell lines (66) makes it a crucial therapeutic target for ovarian cancer. However, the detailed mechanisms by which HDAC7 contributes to the maintenance of the tumor stem cell phenotype have not been investigated. A discovery was reported that valproic acid has the ability to decrease the HDAC7 expression in human ovarian cancer cells when choosing therapeutic medications (67).

### 4.5 Breast cancer

At present, breast cancer has surpassed lung cancer as the most frequently diagnosed tumor among females (52), with the most malignant type being triple-negative breast cancer. In breast cancer tissue, the level of HDAC7 mRNA was higher compared to normal breast tissue (11). In serum samples, HDAC7 protein was significantly higher in patients with recurrent breast cancer compared to non-recurrent breast cancer (68). Furthermore, HDAC7 exhibited high expression in breast cancer stem cells (66). These findings indicate an association between HDAC7 and breast cancer. Through the deacetylation of HSP70 K246, HDAC7 could hinder autophagic cell death, thereby promoting the survival of cancer cells and the emergence of drug resistance in mechanistic investigations (11). In another research, overexpressed HDAC7 was demonstrated to maintain the breast cancer stem cell phenotype by regulating histone 3 lysine 27 acetylation and enhancing transcriptional activity of super-enhancer-associated genes in breast CSCs (32). These findings provide additional theoretical support for the research and development of HDAC7-specific inhibitors. However, two studies showed conflicting results regarding the expression of HDAC7 in triple-negative breast cancer, where it was observed to be high and low, with both expressions associated with poor prognosis (69, 70). The discrepancies in results may be attributed to differences in sample size and individual heterogeneity.

### 4.6 Choroidal melanoma

The most prevalent type of uveal melanoma is choroidal melanoma. Currently, therapeutic regimens for choroidal melanoma are primarily referenced to cutaneous melanoma (71), but the treatment effect is less than half that of cutaneous melanoma. Additionally, there are no effective therapeutic agents for metastatic melanoma. Therefore, it is necessary to find potential therapeutic targets for the treatment of choroidal melanoma. *In vitro* and *in vivo* experiments, HDAC7 was found to be upregulated in choroidal melanoma and demonstrated its interaction with c-myc to affect the growth and migration of tumor cells (36). Previous research indicates that β-catenin plays a crucial role in the HDAC7/c-myc signaling pathway throughout the progression of tumors. However, the relationship between HDAC7 and β-catenin is unclear and needs experiments to be demonstrated in choroidal melanoma.

### 4.7 Hematologic tumors

HDAC7 has two opposite expression profiles in hematologic tumors. In patients diagnosed with acute (72) and chronic (73) lymphoblastic leukemia, this gene is found to be highly expressed. However, in B lineage-derived malignancies such as diffuse large B cell lymphoma (74) and acute lymphoblastic leukemia derived from B-cell progenitors, especially in the infant t (4, 11) phenotype (40), the gene is down-regulated. Both overexpression and down-regulation expressions of HDAC7 this gene are associated with poor prognosis. However, the former lacks relevant molecular mechanism research. In contrast, for the latter, a molecular mechanism manifests that HDAC7 suppresses cell oncogenicity by downregulating the expression of c-myc oncogenes and promoting the expression of genes related to apoptosis (75). Consequently, HDAC7 can exhibit both oncogenic and anti-carcinogenic effects in different hematological malignancies.

### 4.8 Clear cell carcinoma

While enhanced glycolysis has been observed to facilitate tumorigenesis in numerous studies on cancer mechanisms, the expression of enzymes involved in mitochondrial metabolism displays greater heterogeneous. Renal cell carcinoma is one of the tumors with significant alterations in mitochondrial metabolism, particularly with a prominent decrease in the expression of mRNA for tricarboxylic acid cycle enzymes (76). Meanwhile, Nam et al. (7) showed that HDAC7 expression was significantly increased at mRNA and protein levels in clear renal cell carcinoma compared to normal kidneys by analyzing the TCGA database. Moreover, HDAC7 inhibits the expression of tricarboxylic acid cycle enzymes by binding to SMAD, thereby having a negative effect on mitochondrial metabolism, which was manifested in the following that induced the respiratory dysfunction of mitochondria and elevated oncogenes to promote tumorigenesis (7). The results indicate that HDAC7 has a crucial function in tumor metabolism and offers understanding into the molecular mechanism of mitochondrial metabolism changes in clear renal cell carcinoma.

## 5 Pharmacological strategies

Currently, the primary focus of research on HDAC7 inhibitors has been in the realm of cancer, although recent findings suggest their regulatory effects extend to the inflammatory conditions and metabolic dysfunction (77). Several potent inhibitors of HDAC7 enzymatic activity, namely TMP195 (78), CHDI-12 (79), CHDI390576 (80), CHDI00484077 (81), and JM63 (82), have been identified. Nevertheless, it is important to mention that these blockers also display inhibitory impacts on additional class IIa HDACs (HDAC4, 5, 9) and even show a certain degree of inhibition towards class I HDACs. In cellular systems, TMP195 caused alterations in the release of chemokines CCL1 and CCL2 from macrophages derived from human monocytes. Additionally, it had a lesser effect on gene expression in lymphocytes compared to Vorinostat, suggesting its potential for selectively inhibiting class IIa HDACs. Additionally, TMP195 demonstrated efficacy in reducing breast tumors and metastases in mice through the recruitment and differentiation of anti-tumor macrophages (83). Recently, the CHDI Foundation released research results on three groups of potent brain-penetrating HDAC4 (22) inhibitors, which have shown great effectiveness in treating Huntington’s Disease. Specifically, compounds CHDI: 12, 390576, and 00484077 demonstrated potent inhibition of HDAC7 as well. However, additional research is required to investigate the pharmacological approaches employed by CHDI in targeting HDAC7 for cancer treatment. JM63 is a constituent of a group of benzoyl hydroxamate inhibitors that have been identified as the most effective HDAC7 inhibitors documented thus far (82). In experiments conducted on cells, JM63 displayed an inhibitory potency that was 10 times stronger than TMP195 against class IIa HDACs. Additionally, it exhibited improved stability in cytochrome P450-rich rat liver microsomes, with a half-life of 230 minutes instead of 46 minutes (22). As a potential treatment for hepatocellular carcinoma, JM63 shows promise as a pharmaceutical agent.

## 6 Future direction

This paper systematically reviews the role of HDAC7 in cancer. The progress of clinical trials for different types of cancers is advancing with the development of additional pan- and selective inhibitors (84). Therefore, it might be possible to specifically focus on HDAC7 for the treatment of cancer. However, there is no doubt that there is still a long way to go.

### 6.1 The different forms of cancer have varying molecular mechanisms involving HDAC7

Currently, studies have shown that HDAC7 has two expression levels in cancer. For instance, it is overexpressed in gastric cancer (51), and colorectal cancer (10), among others, while it has low expression in pro-B ALL (40). Both of its aberrant expressions exert oncogenic effects in cancer. Additionally, HDAC7 can act as a mediator regulated by miRNAs, playing a role in cancer progression (11, 37, 39, 46). Considering that aberrant expression of HDAC7 correlates with the severity and progression of various cancers, it is possible for it to be a potential biomarker or target. However, making HDAC7 a targeted therapeutic site requires a more precise understanding of the molecular mechanisms that operate in different types of cancer.

### 6.2 The cellular functions of HDAC7 depends on its distribution in cells

Recent studies have been showed that HDAC7 promotes tumorigenesis by promoting tumor proliferation and metastasis (9, 36), inhibiting drug entry into cells to generate drug resistance (10, 85), suppressing autophagy (11, 86, 87), and many other actions. However, HDAC7 has different biological functions in different cell types, depending on its distribution within cells. For example, it ensures proper B-cell expression during B-cell development (3), p However, HDAC7 has different biological functions in different cell types, depending on its distribution within cells. For example, it ensures proper B-cell expression during B-cell development (4) and maintains the proper functioning of the TCA cycle in mitochondrial metabolism (7). Additionally, a comprehensive knockdown of HDAC7 during embryonic development can lead to embryonic lethality (6), among other effects.

### 6.3 Potential transcriptional targets for HDAC7

The increasing range of interacting partners suggests a diverse array of potential transcriptional targets for HDAC7.Nevertheless, most biologically significant targets for HDAC7 have thus far been found to be MEF2-dependent promoters. Therefore, it is imperative to conduct comprehensive inquiries to discover genes that are not dependent on MEF2 but could be controlled by HDAC7.

### 6.4 Selective inhibitors of HDAC7 for the treatment of tumors

Balancing the negative role of HDAC7 in tumors with its role in normal physiological processes is key to the future use of HDAC7 inhibitors for tumor treatment. Thorough examinations regarding the function of HDAC7 in the formation of tumors aid in the investigation of inhibitors that specifically target HDAC7. To achieve the objectives, it is crucial to utilize tissue-specific reduction and thorough mechanistic investigations on the effects of selective inhibitors of HDAC7 both in laboratory settings and within living organisms. Given that HDAC7 exerts oncogenic effects through the same signaling pathway in multiple tumors, it is possible for the same HDAC7 inhibitor to be used as a targeted therapeutic agent for numerous tumors in the future.

## 7 Limitations

Furthermore, we noted that this review had some limitations (1). It is also important to critically evaluate the action of HDAC7 as an epigenetic regulator and the relationships with inflammatory genes. Given the limited space available, our focus will solely be on elucidating the involvement of HDAC7 in cancer. (2) The role of HDAC7 in cancer may have been overlooked. (3) While there is a possibility of undiscovered mechanisms in the future, numerous mechanisms of HDAC7 are still unidentified in this article.

## Author contributions

CL: Writing – original draft, Writing – review & editing. DZ: Validation, Writing – review & editing. XP: Validation, Writing – review & editing. SL: Conceptualization, Resources, Writing – review & editing.
